# Zoonoses and Wildlife: One Health Approach

**DOI:** 10.3390/ani12040480

**Published:** 2022-02-15

**Authors:** David González-Barrio

**Affiliations:** Parasitology Reference and Research Laboratory, Spanish National Centre for Microbiology, Health Institute Carlos III, Ctra. Majadahonda-Pozuelo Km 2, Majadahonda, 28220 Madrid, Spain; dgonzalezbarrio@gmail.com or david.gonzalezb@isciii.es

Throughout history, wildlife has been an important source of infectious diseases transmissible to humans. Today, zoonoses with a wildlife reservoir constitute a major public health problem, affecting all continents. The importance of such zoonoses is increasingly recognized, and the need for more attention in this area is being addressed. The total number of zoonoses is unknown; some 1415 known human pathogens have been catalogued, and 62% are of zoonotic origin [1]. Over time, more and more human pathogens are found to be of animal origin. Moreover, most emerging infectious diseases in humans are zoonoses. Wild animals seem to be involved in the epidemiology of most zoonoses, and serve as major reservoirs for the transmission of zoonotic agents to domestic animals and humans [2]. The concept of the ‘One Health’ approach—involving collaboration between veterinary and medical scientists, policymakers, and public health officials—is necessary in order to foster joint cooperation and control of emerging zoonotic diseases [3]. Zoonotic diseases caused by a wide range of arthropods, bacteria, helminths, protozoans, and viruses can cause serious and even life-threatening clinical conditions in animals, with a number of them also affecting the human population due to their zoonotic potential.

The aim of the present Special Issue is to cover recent and novel research trends in zoonotic diseases in wildlife, including the relevant topics related to wildlife, zoonosis, public health, emerging diseases, infectious diseases, and parasitic diseases. 

A total of 12 papers have been contributed by 96 authors from 14 countries to this issue, comprising 10 research articles, 1 communication, and 1 brief report (Figure 1). The number of specimens studied in this issue amounts to 5132, including wild animals, wild animals kept in captivity, domestic animals, and ticks; even human samples have been analyzed. More than 50 different species—including wild and domestic ungulates (e.g., red deer, roe deer, fallow deer, chamois, mouflon, European bison, wild boar, sheep, goat, cattle), wild carnivores (e.g., wolf, Eurasian lynx, Eurasian badger, coypu, beech marten, golden jackal), micromammals (e.g., yellow-necked field mouse, long-tailed field mouse, European water vole, white-toothed shrew, garden dormouse, common vole, house mouse, western Mediterranean mouse, black rat, Eurasian red squirrel), non-human primates (the genera *Cebuella, Cercocebus, Cercopithecus, Eulemur, Hylobates, Lemur, Macaca, Mandrillus, Saimiri, and Varecia*), turtles (e.g., *Testudo hermanni*, *T. h. boettgeri*, *T. graeca*, and *T. marginata*), bats (the families Pteropodidae, Emballonuridae, Rhinolophidae, Hipposideridae, and Vespertilionidae), and ticks (*Ixodes ricinus*, *Dermacentor marginatus*, *Hyalomma marginatum*)—are included. Regarding the zoonotic pathogens represented in this issue, the presence of or exposure to 17 different pathogens—including viruses [4] (West Nile virus), bacteria [5,6,7,8,9,10,11,12,13] (*Anaplasma phagocytophilum, Coxiella burnetii, Helicobacter pylori, H. suis, Mycobacterium tuberculosis* Complex, *Salmonella* sp., and *Leptospira interrogans* sensu stricto), and parasitic protists [14,15] (e.g., *Cryptosporidium* spp., *Giardia duodenalis*, *Blastocystis* sp., *Enterocytozoon bieneusi*, *Entamoeba histolytica*, *Entamoeba dispar*, *Balantioides coli*, *Troglodytella* spp., *Leishmania* spp.)—are presented.

The study of zoonotic pathogens present in wildlife mainly involves serological and/or molecular analyses, among others, for their detection, which is somewhat costly due to the difficulty in obtaining the necessary samples for analysis and ensuring that they are of high quality [16]; therefore, samples are often obtained from wild animals kept in captivity or in rescue centers [7,14]. In addition, the study of parasites involves searching for them, or their DNA—mainly in the feces of animals. In remote areas or resource-poor settings where the cold chain cannot be maintained, preservation and conservation of biological specimens—including fecal samples—is a challenge; for this reason, Köster et al. [15] evaluated the suitability of filter cards for the long-term storage of fecal samples of animal and human origin that were positive for the diarrhea-causing protozoan parasites *Giardia duodenalis* and *Cryptosporidium hominis*. For this purpose, three commercially available Whatman^®^ filter cards were comparatively evaluated: the FTA^®^ Classic card, the FTA^®^ Elute Micro card, and the 903 Protein Saver card. *Giardia duodenalis* (*n* = 5)- and *C. hominis* (*n* = 5)-positive human stool samples were used to impregnate the selected cards at selected storage times (1 month, 3 months, and 6 months) and temperatures (−20 °C, 4 °C, and room temperature). Data presented by Köster et al. [15] demonstrate that Whatman^®^ cards are a cost-effective option for the preservation and long-term storage (up to six months) of fecal samples under a wide range of temperatures (from −20 °C to room temperature), without compromising their biospecimen stability and suitability for molecular-based diagnostic methods. Indeed, Whatman^®^ cards enable the molecular detection and genotyping of common diarrhea-causing enteric protozoan parasites, including *C. hominis* and *G. duodenalis*.

A significant proportion of wildlife studies are carried out in conservation centers—such as zoos—but also in wildlife rescue centers. Monitoring of infections that may be transmitted to humans by animals in wildlife rescue centers is very important in order to protect the staff engaged in rehabilitation practices. Casalino et al. [7] investigated the occurrence of non-typhoidal *Salmonella* in tortoises housed in a regional wildlife rescue center in Apulia, Southern Italy, to assess the presence of *Salmonella* serovars that may pose a risk to operators involved in wildlife management. *Salmonella* may be a natural inhabitant of the intestinal tracts of turtles, rarely causing disease in turtles. This may represent a potential risk for humans, increasing the sanitary risk for operators in wildlife rescue centers. Casalino et al. [7] tested 69 adult turtles (*Testudo hermanni*, *T*. *h*. *boettgeri*, *T*. *graeca*, and *T*. *marginata*); the distribution of *Salmonella* spp. was significantly higher in *T*. *hermanni* than in other species. Two different *Salmonella* species (*S*. *enterica* and *S*. *bongori*) three *S*. *enterica* subspecies (*enterica*, *diarizonae*, and *salamae*), and five different serovars (Hermannswerder, Abony, Ferruch, Richmond, and Vancouver) within the group *S*. *enterica* subspecies *enterica* were identified. Most of the detected *Salmonella* types may represent a potential risk to public health. Reducing turtles’ stress in order to minimize *Salmonella* shedding, as well as adopting correct animal husbandry procedures and hygiene techniques, may be useful to minimize the risk of transmission of *Salmonella* to humans. In particular, the adoption of gloves to manage turtles is a relevant preventive measure. Nevertheless, the greater measure of prevention is information and education on the potential sanitary risks of each professional figure involved in wildlife management.

On the other hand, little information is currently available on the epidemiology and zoonotic potential of parasitic and commensal protist species in captive non-human primates (NHPs). Köster et al. [14] investigated the occurrence, molecular diversity, and potential transmission dynamics of parasitic and commensal protist species in a zoological garden in southern Spain. The prevalence and genotypes of the main enteric protist species were investigated in fecal samples from NHPs (*n* = 51), zookeepers (*n* = 19), and free-living rats (*n* = 64) via molecular (PCR and sequencing) methods between 2018 and 2019. The presence of *Leishmania* spp. was also investigated in tissues from sympatric rats using PCR. *Blastocystis* sp. (45.1%), *Entamoeba dispar* (27.5%), *Giardia duodenalis* (21.6%), *Balantioides coli* (3.9%), and *Enterocytozoon bieneusi* (2.0%) (but not *Troglodytella* spp.) were detected in NHPs. *Giardia duodenalis* (10.5%) and *Blastocystis* sp. (10.5%) were identified in zookeepers, while *Cryptosporidium* spp. (45.3%), *G. duodenalis* (14.1%), and *Blastocystis* sp. (6.25%) (but not *Leishmania* spp.) were detected in rats. *Blastocystis* ST1, ST3, and ST8, along with *G. duodenalis* sub-assemblage AII, were identified in NHPs, and *Blastocystis* ST1 was identified in zookeepers. In rats, four *Cryptosporidium* (*C. muris*, *C. ratti*, and rat genotypes IV and V), one *G. duodenalis* (assemblage G), and three *Blastocystis* (ST4) genetic variants were detected. These results indicate high exposure of NHPs to zoonotic protist species. In conclusion, strong evidence of the occurrence of zoonotic *Blastocystis* transmission between NHPs and their handlers was provided, despite the use of personal protective equipment and the implementation of strict health and safety protocols. Free-living sympatric rats are infected by host-specific species/genotypes of the investigated protists, and seem to play a limited role as a source of infections to NHPs or humans in this setting.

Interactions taking place between sympatric wildlife/livestock/humans may contribute to interspecies transmission of pathogens [17]—this is the case of the *Mycobacterium tuberculosis* complex [18]. Mycobacteria can cause medically and socioeconomically significant diseases, including several non-tuberculous infections and tuberculosis, and are considered a One Health challenge due to their impact on public and animal health. These microorganisms are maintained and shared between the environment, domestic and wild animals, and humans. In this Special Issue, two studies are related to the interaction between domestic and wild species and the detection of mycobacteria in wild species such as badgers. Varela-Castro et al. [6] characterized the interactions that take place between several wild mammals and cattle via camera-trapping in order to provide insights into the dynamics of mycobacterial transmission opportunities in the environment of cattle farms located in Atlantic habitats in the northern Iberian Peninsula. Camera traps were set during a one-year period in cattle farms with a history of tuberculosis and/or non-tuberculous mycobacteriosis. A total of 1293 visits were recorded during 2741 days of camera observation. Only 23 visits showed direct contacts with cattle, suggesting that mycobacterial transmission at the wildlife–livestock interface occurs mainly through indirect interactions. Results showed that cattle pastures represented the most appropriate habitat for interspecies transmission of mycobacteria, and badgers’ latrines appear to be a potential hotspot for mycobacterial circulation between badgers, wild boars, foxes, and cattle. According to both previous epidemiological information and the interaction patterns observed, wild boars, badgers, foxes, and small rodents are the species or groups most often in contact with livestock and, thus, may be the most involved in the epidemiology of mycobacteriosis in the wildlife–livestock interface in this area. As Valera-Castaro et al. [6] pointed out in their work, the badger and its latrines are a hotspot for interspecies transmission—both domestic and wild; more specifically, Blanco Vázquez et al. [9] investigated the prevalence, spatial distribution, and temporal distribution of tuberculosis in 673 free-ranging Eurasian badgers (*Meles meles*) in Asturias (Atlantic Spain) between 2008 and 2020. The study’s objective was to assess the role of badgers as a tuberculosis reservoir for cattle and other sympatric wild species in the region. Serum samples were tested in an in-house indirect P22 ELISA to detect antibodies against the *Mycobacterium tuberculosis* complex (MTC). In parallel, data on MTC isolation and single intradermal tuberculin test results were extracted for cattle that were tested and culled as part of the Spanish National Program for the Eradication of Bovine Tuberculosis. A total of 27/639 badgers (4.23%) were positive for MTC based on bacterial isolation, while 160/673 badgers (23.77%) were found to be positive with the P22 ELISA. The rate of seropositivity was higher among adult badgers than sub-adults. The authors found that the tuberculosis status of badgers in Asturias during 2008–2020 was associated with the tuberculosis status of local cattle herds, and results could not determine the direction of possible interspecies transmission, but they were consistent with the idea that the two hosts may exert infection pressure on one another. Both studies highlight the importance of monitoring this multi-host infection and disease in wildlife during epidemiological interventions in order to optimize outcomes under the One Health concept.

Deadly emerging and re-emerging zoonotic pathogens are transmitted mostly from wildlife reservoirs to humans or other animals during spillover events, with or without a vector intervention. In this special issue, two papers are included in which vector-borne zoonotic pathogens were studied. Ain-Najwa et al. [4] highlight the first evidence of West Nile virus (WNV) infection—a mosquito-borne virus—in Malaysian macaques and bats. Of the 81 macaques from mangrove forests sampled, 24 of the long-tailed macaques were seropositive for WNV, indicating that they were exposed to the virus; meanwhile, 5 out of 41 bats that were found in the caves from northern Peninsular Malaysia showed susceptibility to WNV. The authors found a high WNV antibody prevalence in macaques and a moderate WNV RNA in various Malaysian bat species, suggesting that WNV circulates through Malaysian wild animals, and that Malaysian bat species may be susceptible to the WNV infection. On the other hand, Grassi et al. [12] researched the genetic variants of *Anaplasma phagocytophilum* (a tick-borne pathogen causing zoonotic disease) in wild ungulates (the leading reservoir species) and feeding ticks (the main vector of infection) from northeastern Italy. Using biomolecular tools and phylogenetic analysis, ecotypes I and II were detected in both ticks (*Ixodes ricinus* species) and wild ungulates. Specifically, ecotype II was mainly detected in roe deer and related ticks, while ecotype I—the potentially zoonotic variant—was detected in *Ixodes ricinus* ticks, and also in wild ungulates. These findings reveal not only the wide diffusion of *Anaplasma phagocytophilum*, but also the presence of zoonotic variants.

Žele-Vengušt et al. [5] analyzed the exposure of free-ranging wild animals to zoonotic *Leptospira interrogans* sensu stricto in Slovenia; for this, blood samples from 249 wild animals between 2019 and 2020 were tested using the microscopic agglutination test for specific antibodies against the *Leptospira* serovars Icterohaemorrhagiae, Bratislava, Pomona, Grippotyphosa, Hardjo, Sejroe, Australis, Autumnalis, Canicola, Saxkoebing, and Tarassovi. Antibodies to at least one of the pathogenic serovars were detected in 77 (30.9%; CI = 25–37%) sera. The proportion of positive samples varied intraspecifically, and was the greatest in large carnivores (86%), followed by mesopredators (50%) and large herbivores (17%). Out of the 77 positive samples, 42 samples (53.8%) had positive titers against a single serovar, while 35 (45.4%) samples had positive titers against two or more serovars. The most frequently detected antibodies were those against the serovar Icterohaemorrhagiae. This study confirmed the presence of multiple pathogenic serovars in wildlife throughout Slovenia. It can be concluded that wild animals are reservoirs for at least some of the leptospiral serovars, and are a potential source of leptospirosis for other wild and domestic animals, as well as for humans.

In their study, Cortez Nunes et al. [13] investigated the presence of *Helicobacter pylori* and *H. suis* DNA in free-range wild boars. *Helicobacter pylori* and *H. suis* are associated with gastric pathologies in humans. Interactions between domestic animals, wildlife, and humans can increase the risk of bacterial transmission between species. Samples of the gastric tissue of 14 free range wild boars (*Sus scrofa*) were evaluated for the presence of *H. pylori* and *H. suis* using PCR. Two samples were PCR-positive for *H. pylori*, and another for *H. suis*. These findings indicate that these microorganisms were able to colonize the stomachs of wild boars, and raise awareness of their putative intervention in the transmission cycle of *Helicobacter* spp..

Finally, this Special Issue includes three articles dealing with the potential role of livestock and wildlife as potential sources of human Q fever. Q fever is a worldwide-distributed zoonosis caused by *Coxiella burnetii*—a small intracellular bacterium belonging to γ-Proteobacteria that infects a wide range of animal species, including mammals, birds, and arthropods. People are infected through inhalation of aerosols contaminated with the bacteria expelled by infected animals during abortion or normal deliveries. Domestic ruminants, sheep, and goats are considered the main reservoirs of the infection and the principal source of human outbreaks. *Coxiella burnetii* has a complex ecology that replicates in multiple host species; however, the role of wildlife in its transmission is poorly understood. Krzysiak et al. [11] examined 523 serum samples obtained from European bison for the presence of specific antibodies in order to assess whether infection occurs in this species, and whether European bison may be an important source of infection in the natural environment, as suggested by historical reports. Only one (0.19%) serum sample was positive in ELISA, and two other samples were doubtful; the only seropositive animal was a free-living bull. This suggests possible transmission from domestic cattle by sharing pastures. The transmission of *C*. *burnetii* into the European bison was rather accidental in the country, and its role as an important wild reservoir is unlikely. In their study, González-Barrio et al. [10] examined spleen samples from 816 micromammals of 10 species, and 130 vaginal swabs from *Microtus arvalis* by qPCR, to detect *C. burnetii* infection and shedding, respectively; 9.7% of the spleen samples were qPCR-positive. The highest infection prevalence (10.8%) was found in *Microtus arvalis*, in which *C. burnetii* DNA was also detected in 1 of the 130 vaginal swabs (0.8%) analyzed. Positive samples were also found in *Apodemus sylvaticus* (8.7%), *Crocidura russula* (7.7%), and *Rattus rattus* (6.4%). Positive samples were genotyped by coupling PCR with reverse line blotting, and a genotype II+ strain was identified for the first time in one of the positive samples from *M. arvalis*, whereas only partial results could be obtained for the rest of the samples. Acute Q fever was diagnosed in one of the researchers who participated in the study, and it was presumably linked to *M. arvalis* handling. The results of the study are consistent with previous findings suggesting that micromammals can be infected by *C. burnetii*. The authors additionally suggest that micromammals may be potential sources to trace back the origin of human Q fever and animal coxiellosis cases in Europe, and might be relevant in the maintenance of wild-type *C. burnetii* strains that can be a matter of concern for animal and human health authorities. Espí et al. [8] investigated the seroprevalence of *C. burnetii* in domestic ruminants and wild ungulates, as well as the current situation of Q fever in humans, in a small region in northwestern Spain, where close contact at the wildlife–livestock–human interface exists, and information on *C. burnetii* infection is scarce. Seroprevalence of *C. burnetii* was 8.4% in sheep, 18.4% in cattle, and 24.4% in goats. Real-time PCR analysis of environmental samples collected in 25 livestock farms detected *Coxiella* DNA in dust and/or aerosols collected in 20 of them. Analysis of sera from 327 wild ungulates revealed lower seroprevalence than that found in domestic ruminants. Exposure to the pathogen in humans was determined by IFAT analysis of 1312 blood samples collected from patients admitted to healthcare centers with Q-fever-compatible symptoms, such as fever and/or pneumonia. Results showed that 15.9% of the patients had IFAT titers ≥ 1/128, suggestive of probable acute infection. This study is an example of a One Health approach with medical and veterinary institutions involved in investigating zoonotic diseases.

Overall, the papers in this Special Issue reveal different perspectives of current research on zoonotic disease and wildlife, from applied field studies to investigations into the intricate mechanisms involved in the interaction between pathogens, wildlife, livestock, and humans. 

## Figures and Tables

**Figure 1:**
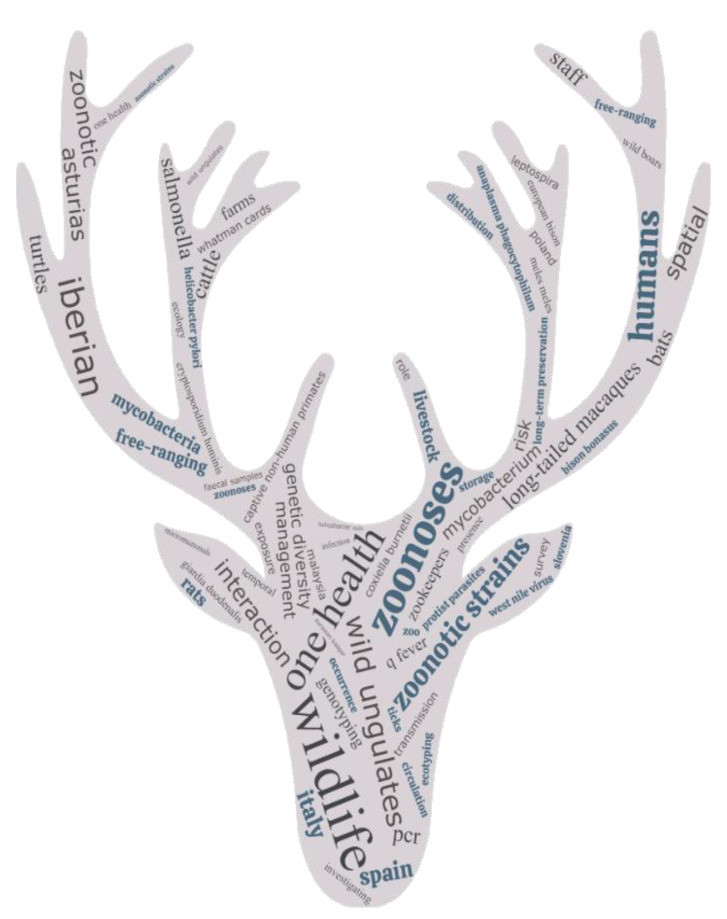
A word cloud created from the titles of every article published in this Special Issue.
